# Prediction model of compensation for contralateral kidney after living-donor donation

**DOI:** 10.1186/s12882-019-1464-1

**Published:** 2019-07-26

**Authors:** Kenji Okumura, Shigeyoshi Yamanaga, Kosuke Tanaka, Kohei Kinoshita, Akari Kaba, Mika Fujii, Masatomo Ogata, Yuji Hidaka, Mariko Toyoda, Soichi Uekihara, Akira Miyata, Akito Inadome, Hiroshi Yokomizo

**Affiliations:** 1 Department of Surgery, Japanese Red Cross Kumamoto Hospital, 2-1-1 Nagamine-minami, Higashi-ku, Kumamoto, 861-8520 Japan; 2 Department of Nephrology, Japanese Red Cross Kumamoto Hospital, Kumamoto, Japan; 3 Department of Urology, Japanese Red Cross Kumamoto Hospital, Kumamoto, Japan

**Keywords:** Kidney transplant donor, Renal function compensation, CT volumetry, Remnant kidney volume

## Abstract

**Background:**

Compensation of contralateral kidney function after living-donor kidney donation is well known, and many predictive factors have been proposed. However, no prediction model has been proposed. This study was performed to establish a tool with which to estimate the degree of compensation of the contralateral kidney after living-donor kidney donation.

**Methods:**

We retrospectively analyzed 133 living donors for renal transplantation in our institution. We defined a favorable compensation as a post-donation estimated glomerular filtration rate (eGFR) at 1 year (calculated by the Chronic Kidney Disease Epidemiology Collaboration equation) of > 60% of the pre-donation eGFR. We analyzed the living donors’ clinical characteristics and outcomes.

**Results:**

The median (range) donor age was 59 (24–79) years, median (range) body mass index was 22.9 (16.8–32.7) kg/m^2^, and median (range) body surface area was 1.6 (1.3–2.0) m^2^. All donors were Japanese, and 73% of the donors were biologically related. The median (range) donor pre-donation eGFR was 108.7 (82–144) ml/min/1.73 m^2^, and the median (range) post-donation eGFR at 1 year was 86.9 (43–143) ml/min/1.73 m^2^. Eighty-six percent of donors had compensatory hypertrophy. In the univariate analysis, age, female sex, history of hypertension, body surface area, and pre-donation eGFR were significantly associated with hypertrophy (*p* < 0.05). In the multivariate analysis, age, female sex, history of hypertension, and ratio of the remnant kidney volume to body weight were significantly associated with hypertrophy (*p* < 0.05). Based on these results, we created a compensation prediction score (CPS). The median (range) CPS was 8.7 (1.1–17.4). Receiver operating characteristic analysis showed strong diagnostic accuracy for predicting favorable compensation (area under the curve, 0.958; 95% confidence interval, 0.925–0.991, *p* < 0.001). The optimal cut-off value of the CPS was 5.0 (sensitivity, 92.0%; specificity, 89.5%). The CPS had a strong positive correlation with the post-donation eGFR (R = 0.797, *p* < 0.001).

**Conclusion:**

The CPS might be useful tool with which to predict a favorable compensation of the contralateral kidney and remnant kidney function. If the CPS is low, careful management and follow-up might be necessary. Further investigations are needed to validate these findings in larger populations.

## Background

Kidney transplantation has been considered a preferred treatment for patients with end-stage renal disease since it provides longer survival substantially and better quality of life than dialysis [1]. As the numbers of patients with end-stage renal disease are increasing, the demand for expansion of the donor pool has been intensified, and living-donor kidney transplantation is one of the solutions for the donor shortage, especially in Japan [2]. Although a large cohort study showed that living-donor kidney donation is safe, donor nephrectomy is still a major procedure associated with potential risks for the donor, including increased a cardiovascular risk and progression to end-stage renal disease in the long term [3].

Compensatory kidney function of the contralateral kidney after donation has been well known, and many predictive factors for post-donation kidney function in kidney donors have been proposed [4–8]. However, few studies have been performed to investigate preoperative factors that might affect the degree of renal recovery after kidney donation [9], and none have been performed to establish a prediction model with which to estimate compensation of contralateral kidney after living donation.

Therefore, this study was performed to establish a prediction model with which to estimate compensation of the contralateral kidney after living-donor kidney donation using preoperative data.

## Methods

We retrospectively analyzed 133 consecutive living kidney donors in our institution from January 2011 to December 2017. All donors were medically fit for donation based on the Japanese donor selection criteria [10]. We used the Chronic Kidney Disease Epidemiology Collaboration (CKD-EPI) equation to calculate the estimated glomerular filtration rate (eGFR) in our cohort [9, 11]. The body surface area (BSA) was calculated based on the DuBois–DuBois formula. All donors underwent a computed tomography (CT) scan, including three-dimensional CT volumetry, during their preoperative evaluation. Three-dimensional CT volumetry was performed using ZIOSTATION 2® (Ziosoft, Tokyo, Japan). We investigated the relationship between allograft function and donor kidney volume using CT volumetry to select the donor’s kidney (right or left) to be transplanted. We analyzed the donors’ clinical characteristics and outcomes and created a prediction model.

### Definition of favorable compensatory

We defined favorable compensatory hypertrophy as a post-donation eGFR at 1 year (calculated by the CKD-EPI equation) of > 60% of the pre-donation eGFR. The cut-off eGFR of 60% was based on a previous study that revealed a typical post-donation eGFR range of 62.5 to 67.0% of the baseline renal function [12].

### Statistical analysis

Statistical analyses were performed using IBM SPSS Statistics 24.0 (IBM Corp., Armonk, NY, USA). Non-parametric analysis was used to compare continuous variables, and Pearson’s chi-square test was used for categorical data. Multivariate analysis was performed with a stepwise logistic regression model. For all statistical analyses, *p* < 0.05 was taken as statistically significant.

## Results

Table 1 shows the characteristics of 133 patients who were assigned to either the hypertrophy or non-hypertrophy group according to their eGFR (CKD-EPI equation). All donors were Japanese, and 73% of the donors were biologically related.

**Table 1:** Characteristics of patients

Variables	Hypertrophy (*n* = 114)	Non-hypertrophy (*n* = 19)	*p*
Age (years)	58 (24–79)	65 (49–73)	0.002
Sex (M:F)	29: 85	17:2	< 0.001
Height (m)	1.57 (1.40–1.83)	1.65 (1.56–1.75)	< 0.001
Weight (kg)	55 (40–87)	69 (52–83)	< 0.001
Hypertension	45 (39)	15 (79)	0.002
History of smoking	34 (30)	3 (16)	0.274
BMI(kg/m^2^)	22.3 (17.8–32.7)	25.0 (16.8–28.5)	0.008
BSA(m^2^)	1.56 (1.30–2.04)	1.72 (1.35–1.96)	0.001
Preoperative Serum Cr (mg/dl)	0.62 (0.37–0.91)	0.81 (0.59–0.94)	< 0.001
Preoperative eGFR (ml/min/1.73m^2^)	110.5 (82–144)	94 (86–136)	< 0.001
Volume of donated kidney (ml)	146 (93.9–251)	169 (116–219)	0.062
RKV (ml)	143 (89.3–237)	161 (105–203)	0.368
RKV/Weight (ml/kg)	2.59 (1.61–3.73)	2.26 (1.70–2.87)	0.001
RKV/BSA (ml/m^2^)	92.8 (62.1–132)	90.7 (62.3–119)	0.298

*BMI* body mass index, *BSA* body surface area, *Cr*: creatinine, *eGFR* estimated glomerular filtration rate, *RKV* remnant kidney volume

Data are n (%) or median (range)

The donors in the hypertrophy group were significantly younger than those in the non-hypertrophy group (mean age, 58 vs 64 years respectively; *p* = 0.020). The median height, body weight (Wt), body mass index (BMI), and body surface area (BSA) were significantly lower in the hypertrophy group than non-hypertrophy group (height: 1.57 vs 1.65 m, *p* < 0.001; Wt: 55 vs 68 kg, *p* < 0.001; BMI: 21.2 vs 22.8 kg/m^2^, *p* = 0.005; and BSA: 1.56 vs 1.76 m^2^, *p* = 0.024, respectively).

The ratio of donors with hypertension was significantly lower in the hypertrophy group than in the non-hypertrophy group (40% vs 74%, respectively; *p* = 0.011). The median preoperative serum creatinine concentration was significantly lower in the hypertrophy group than in the non-hypertrophy group (0.62 vs 0.81 mg/dl, respectively; *p* < 0.001). The median preoperative eGFR was significantly higher in the hypertrophy group than in the non-hypertrophy group (110.5 vs 94.0 ml/min/1.73 m^2^, respectively; *p* < 0.001).

Although the remnant kidney volume (RKV), donated kidney volume, and ratio of the RKV to BSA were not significantly different between the groups, the ratio of RKV to Wt (RKV/Wt) was significantly higher in the hypertrophy group than in the non-hypertrophy group (2.59 vs. 2.26 ml/kg, respectively; *p* = 0.001).

### Multivariate analysis

We performed a stepwise logistic regression analysis using these covariates (Table 2). The multivariate analysis showed that age, hypertension, and the RKV/Wt ratio were significantly associated with hypertrophy (*p* < 0.05).

**Table 2:** Multivariate analysis of predicting compensation

Variables	β (S.E.)	Odds ratio (95% sCI)	*p*
Age	− 0.94 (0.41)	0.910 (0.840–0.986)	0.021
Sex	4.23 (1.03)	68.7 (9.2–512)	< 0.001
History of HTN	2.07 (0.90)	7.95 (1.36–46.4)	0.021
RKV/Weight (per 0.1)	0.38 (0.118)	1.46 (1.16–1.84)	0.001

*HTN* hypertension, *RKV* remnant kidney volume, *CI* confidence interval

Based on this result, we created the following prediction model:

Compensation prediction score (CPS) = 4 × (RKV)/Wt − (age)/10 (+ 4.5 if female) (+ 2.2 if no history of hypertension).

### Diagnostic value of the prediction model

The median CPS was 8.7 (range, 1.1–17.4). Receiver operating characteristic analysis showed strong diagnostic accuracy for predicting hypertrophy (area under the curve, 0.958; 95% confidence interval, 0.925–0.991; *p* < 0.001).

The optimal cut-off value of the CPS was 5.0 (sensitivity, 92.0%; specificity, 89.5%).

**Fig. 1:**
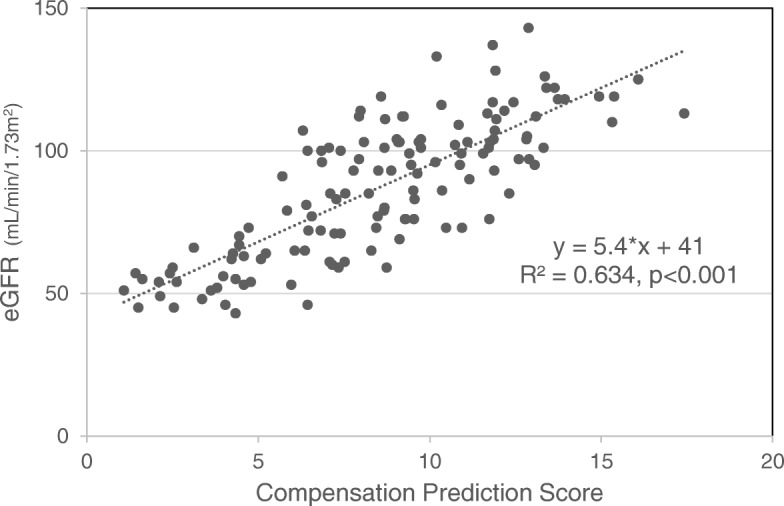
Correlation of eGFR and Compensation Prediction Score. The X-axis represents Compensation Prediction Score. The Y-axis represents the eGFR at one-year after donation. The eGFR and hypertrophy prediction score are positively correlated

The CPS had a strong positive correlation with the post-donation eGFR (R = 0.797, *p* < 0.001) (Fig. 1). According to the correlation analysis, the post-donation eGFR was estimated as follows: Post-donation eGFR = 5.4 × CPS + 41.

## Discussion

The risk of end-stage renal disease after living donation is a major concern, highlighting the importance of determining how to optimize the selection of donors as well as how to achieve the best post-transplant outcomes [13]. In the present study, age, sex, hypertension, and the RKV/Wt ratio were significant preoperative predictors for patients who lost > 40% of their eGFR at 1 year after kidney donation.

As Shinoda et al. [9] reported, the BMI and RKV/BSA ratio are predictive factors for patients who have lost > 30% of their eGFR at 1 year after donation. Compensation of residual kidney function ranges from 60 to 70% on average [9, 14–16], as reported by Blantz et al. Thus, all donors would have, on average, > 30% less “renal reserve,” making a lower post-donation eGFR an important renal risk factor [15, 17]. We aimed to determine whether identification of donors with a below-average recovery of eGFR is more important than identification of donors with an above-average recovery of eGFR. Therefore, we established 60% as the cut-off to categorize donors.

The BSA-adjusted RKV is an independent predictor of the eGFR at 1 year in living kidney donors as demonstrated by Yakoubi et al. [18]. With respect to recipient outcomes, the RKV/Wt ratio has been suggested as an important factor related to allograft function [19, 20]. Although a lower RKV/Wt ratio can cause hyperfiltration and subsequent proteinuria [20, 21], Song et al. suggested that a RKV/Wt ratio of < 2.0 ml/kg did not affect the eGFR in donors but was associated with more severe proteinuria at 1 year after surgery [16]. Although they found no significant differences in the RKV/Wt ratio, their findings suggest deterioration of kidney function based on proteinuria; this in turn suggests that a lower RKV/Wt ratio might affect hyperfiltration and subsequence to decrease “renal reserve.”

For age and hypertension, Denic et al. investigated the relationships among kidney risk factors and they showed that mild hypertension and aging are risk factors for underlying abnormalities such as nephrosclerosis and nephron hypertrophy in donors [22]. In addition, aging cause some of the functional and structural changes [23]. Based on the current understanding of kidney aging, people of advanced age have less reserve of kidney function when they tend to develop CKD and are at higher risk of acute kidney injury [24, 25]. Although the prevalence of hypertension also increases with aging, glomerular hypertrophy has been identified as an integral feature of hypertensive nephropathy and seems to precede rather than to compensate for glomerulosclerosis [26]. Shiraishi et al. reported that factors such as age, male sex, BMI and hypertension were correlated strongly with declining renal function [27]. These study support for our result that age and hypertension are risk factors of unfavorable compensation at 1 year after kidney donation.

Regarding smoking and gender, Shiraihi et al. reported that current smoking was inversely correlated with the presence of renal function decline [27]. Considering the fact that the smoking rate is higher in men than women in Japan [28], the negative effects with respect to compensation might be more closely related to smoking effects than sex-related effects. However, the potential effects of sex on kidney injury are controversial. The hormonal modulation could replicate the effects of sex on the course of kidney disease, which suggested that sex hormones could be important determinants of the greater susceptibility of males than females to progressive kidney injury [29–32]. In our study, female sex had a strong protective effect against kidney hypertrophy after kidney donation, which might be due to the protective effects against kidney injury despite the lack of a difference in smoking. Further investigations in larger populations are needed.

This study has some limitations. This study is a retrospective and performed at a single-institution, and the sample size of the patients was relatively small. We evaluated one-year outcome and one-year follow-up was short and don’t know how this affect in the long term, but this study was performed to identify patients without compensation of kidney function of the contralateral kidney. Our scoring system would be useful for this purpose. We used eGFR in this study since this is less invasive and widely used to follow up donors, but other modality measuring eGFR might have better analysis regarding to assess residual kidney function.

## Conclusion

The CPS might be a useful tool with which to predict hypertrophy of the contralateral kidney and remnant kidney function. If the CPS is low, careful management and follow-up might be necessary. Further investigations are needed to validate our findings in larger populations.

## Data Availability

The datasets analyzed during the current study are available from the corresponding author on reasonable request.

## Abbreviations

BMI: Body mass index
BSA: Body surface area
CKD-EPI: Chronic Kidney Disease Epidemiology Collaboration
CT: Computed tomography
eGFR: Estimated glomerular filtration rate
HTN: Hypertension
RKV: Remnant kidney volume
Wt: body weight
